# Cosmic coding and transfer storage (COSMOCATS) for invincible key storage

**DOI:** 10.1038/s41598-023-35325-y

**Published:** 2023-05-30

**Authors:** Hiroyuki K. M. Tanaka

**Affiliations:** 1 University of Tokyo, Tokyo, Japan; 2 International Virtual Muography Institute (VMI), Global, Tokyo, Japan

**Keywords:** Particle physics, Experimental particle physics, Electrical and electronic engineering, Computational science, Computer science, Mathematics and computing, Information technology

## Abstract

Thus far, a perfectly secure encryption key storage system doesn’t exist. As long as key storage is connected to a network system, there is always a chance that it can be cracked. Even if storage is not continually connected to a network system; it is repeatedly necessary for an individual to access storage to upload and download the data; hence there is always a loophole with every conventional encryption key storage system. By utilizing the penetrative nature of cosmic-ray muons, the COSMOCAT (Cosmic coding and transfer) technique may tackle this problem by eliminating the requirement for any network connection to data storage. COSMOCAT was invented as a post quantum key generation and distribution technique for wireless near field communication. However, in its first stage of development, COSMOCAT relied on standard comparators and Global Positioning System (GPS) or other Global Navigation Satellite Systems (GNSS) for key generation. Temporal jitters of the signals outputted from comparators and frequency fluctuations in GPS-disciplined oscillators degraded the key strength and the efficiency of both the key generation and distribution. New strategies are tested in this paper to improve these factors. As a result, the key strength and the key authenticating rate limit are respectively improved by 4 orders of magnitude and more than 5 orders of magnitude. As a consequence, it has become possible to propose a practical methodology for a new key storage and authentication strategy which has the potential to be an impregnable defense against any kind of cyber/physical attack to data storage. Practical applications of COSMOCATS-based symmetric-key cryptosystems to an electronic digital signing system, communication, and cloud storage are also discussed.

## Introduction

While quantum computing promises unprecedented speed and power for processing data, it also poses new risks to public key encryption. As this technology advances over the next decade, its new capabilities could be exploited to break standard encryption methods that are widely used to protect customer data, complete business transactions, and provide secure communications. New schemes are urgently required to prepare for the transition to post-quantum cryptography so that potentially vulnerable data, algorithms, protocols, and systems can be properly protected.

With all architectural solutions using cryptographic keys stored in cloud storage, there is a limit to the degree of security assurance that the cloud consumer can expect to get; this is due to the fact that the logical and physical organization of the storage resources are entirely under the control of the cloud provider^1^. In particular, the cloud service client’s assets are susceptible to key leakage and key corruptions. Loss of a client’s encryption key results in severe security issues^2^. Potential attackers may have the power to generate a new re-encryption key from stored re-encryption keys^3^. Even if the keys are protected from cyberattacks, the storage system is still vulnerable since, unless it's physically protected inside an underground safe without an entrance, there is always a possibility that a third person could physically access and steal the storage assets or the storage itself. Therefore, an uncrackable encryption key storage system cannot exist in the conventional sense and thus, several security protocols have been established to minimize data storage security weaknesses^4^. However, if we could safely send keys (without using any physical route such as Ethernet cables, Wi-Fi, optical fibers, Bluetooth etc.) from an internet connected server to storage facilities that are completely concealed inside an inaccessible underground safe, (totally isolated from the outside environment), then we could realize an invincible data storage system which would be entirely safe from security threats since it would be impossible to conduct either physical or cyber-attacks on this system. Security of the key storage system crucial in the fields of vehicle social networks^5^, blockchain-based data sharing systems^6^, multiround transfer learning and modified generative adversarial networks^7^, and consumer-centric internet of medical things for cyborg applications^8^.

In the current scheme of smartphone devices, the keys are stored in non-volatile memory (NVM). However, due to their robust electrical nature, these NVMs are susceptible to various physical attacks. In many cases, to protect against physical attacks, protection circuitry and tampering detection mode need to be continually activated; hence a constant power supply is required^9^. For this reason, one of the most popular areas in recent hardware security research is focusing on replacing an NVM key storage embedded in e.g., smart cards with physically unclonable functions (PUF)^10–12^. PUF realizes hardware-based security mechanisms, and was initially developed by Pappu et al.^13^. It facilitates tamper detection, encryption and device fingerprinting features that can be used for identification and authentication of the device; thus, PUF ultimately eliminates the need to store the secret keys in the NVM of the devices^13–15^. However, the PUF's response can be altered by the following factors: temperature changes, aging, drift, electromagnetic interactions, and other noise sources; thus, several types of corrections are required^16–21^. Another developmental direction is using Bluetooth technology that enables much simpler acquisition of link keys^22^. However, it is physically possible for attackers to replace the actual Bluetooth adapter with a malicious adapter to retrieve the stored link keys. If the keys are stored in plain text in the smartphone memory, the attacker can obtain unrestricted access to all Bluetooth services on the targeted smartphone^23^. As long as the key storage is located at accessible locations, physical attacks cannot be avoided with 100% certainty. On the other hand, since COSMOCAT uses highly penetrative comic-ray muons as key generators, and these muons pass through both detectors located above and below massive materials, these muogenic keys could be generated in two locations at the same time; for example, in an above ground data storage facility and in a non-accessible place such as inside an underground safe or submarine. This strategy would eliminate the need to store secret keys in the accessible aboveground device. Moreover, since the randomness of the arrival time of cosmic-ray muons are not affected by terrestrial environments, there is no drift effect in COSMOCAT, and since the muons used for COSMOCAT is highly energetic, the key generation rate is not strongly affected by variations in ambient temperature and electromagnetic field.

Quantum key distribution (QKD) has been proposed as a guaranteed and secure method to share private keys between the sender and the receiver. However, in 2016, Yuen reviewed the problems associated with QKD security^24^. Accordingly, a white paper from the National Cyber Security Centre (NCSC), UK, proposed a halt to QKD development^25^ due to the following concerns regarding QKD:

A. Security research such as authentication is not incorporated into QKD research
B. In order to build a network with QKD, it is necessary to set up a relay point which relies on classical physics, so it is not unconditionally secure.
C. Attack methods that may target QKD devices are also being researched, but such research does not guarantee complete security. An unknown loophole may exist.
D. Updating QKD equipment requires hardware replacement.

QKD may offer highly secured key distribution in principle, but in practice, it cannot be used for upgrading the security of data storage. From the point of view of practicality, there might be more value in researching an alternative methodology with stronger security potential which can be retrofitted to currently available systems.

COSMOCAT has been invented as a post-quantum key generation and distribution scheme for near field communication under a common key cryptosystem^26^. In COSMOCAT, cosmic-ray muons are used as a natural resource to generate random numbers. As long as the same specific muons pass through the detectors, by recording the arrival time of those muons and using the timestamps as random data for cryptographic keys, each detector can independently generate the same secret keys without having to exchange the keys to one another. The process is as follows: by recording the muon's arrival times, sequences of true random numbers (TRNs) are obtained. If the sender and receiver are close enough (≤ 10 m) to each other to detect the same muons, then after subtraction of the muon's TOF between the sender and the receiver, identical numerical sequences can be independently taken from data recorded at both of the COSMOCAT sensors located at the sender and the receiver locations. Thus, both the sender and the receiver can hold the same TRN sequences (called cosmokeys) without having to physically exchange the data. The number of digits that can be used for cosmokeys depends on precision of measuring the moment the cosmic muons are detected.

Pseudo random number generators are cost-effective, but if the algorithm that generates the numbers is stolen by hackers, keys can be easily decrypted if attackers can correctly predict future number sequences. COSMOCAT essentially realizes a method to obtain a one-time pad which is independently known by only the sender and the receiver and which cannot be cracked; thus, COSMOCAT qualifies as an information-theoretic security technique. Nevertheless, in its first iteration, COSMOCAT was using GPS-disciplined oscillators (GPS-DO) for time synchronization to generate keys for encoding. GPS and other GNSS have enabled widespread adoption of Positioning, Navigation, and Timing (PNT) services in many applications across modern society. Comparators are used for binalizing the muon signals and GPS/GNSS systems are used for synchronizing the detectors of these individuals. However, the following drawbacks exist when using the regular comparators and a GPS-based timing system:

A. Regular comparators have relatively large temporal jitters in the binalizing timing of the detector's output signals.
B. GPS-DOs are usually steered to the GPS time every few minutes, thus the resultant frequency output is strongly influenced by frequency fluctuations of local oscillators. These fluctuations degrade the performance of COSMOCAT.
C. GPS signals are unavailable in underground environments.
D. Since GPS signals are low-power and unencrypted, there is a vulnerability to interference, jamming, and spoofing. While interference arises from unintentionally produced RF waveforms that raise the effective noise in the receiver processing, jamming is caused by intentionally producing these RF waveforms. Spoofing is caused by unintentional, intentional or malicious activities which generate RF waveforms that mimic true signals to cause a range of effects: from incorrect PNT outputs to receiver malfunction. Therefore, GPS-dependent disruption or interference with PNT systems has the potential (as it was used in its first iteration) to have adverse impacts on the otherwise excellent security of COSMOCAT.

There are several techniques which could be employed to better enhance the independency and the accuracy of timing used in COSMOCAT. These techniques include (1) direct universal time coordinate (UTC) dissemination e.g., via optical fibers, (2) atomic clocks, and (3) cosmic time calibration (CTC)^27^. All of these techniques offer timing precision of a few ns or better, however they are either (1) costly or (2) have a tendency to drift after being used for long periods of time. Amongst all of these techniques, the cheapest and the most stable solution is CTC. For the second iteration of COSMOCAT described in this work, (A) we replaced the comparator used in the prior work with the constant fraction discriminator (CFD) and improved the key strength and key generation efficiency by 4 orders of magnitude and more than 5 orders of magnitude, respectively. (B) Based on this key generation efficiency, an unbreakable underground key storage security system was designed using a combination of COSMOCAT and CTC by employing one of the major features of COSMOCAT: the penetrative nature of muons (which can automatically generate cosmokeys) which can reach locations deep underground. (C) A protocol for authentication of the encrypted data with the keys stored in the storage in the underground safe is proposed with an introduction on how this could be applied to a digital signing system in cryptocurrency operations. In the current work, a drastic reduction of this timing uncertainty was attempted by introducing a constant fraction discriminator (CFD) and RG-50 co-axial cables instead of the regular comparator and GPS/GNSS systems used in the previous version of COSMOCAT.

## Results

In this section, the basic principles of COSMOCATS along with two key results (1. improvements in key strength and key generation and 2. distribution efficiency) will be outlined. Then the newly created COSMOCATS (the procedures followed at the sender's side and the storage side) will be discussed in more detail in the next section.

COSMOCAT has two features. (1) COSMOCAT can duplicate and triplicate numerical sequences of TRNs in different locations. If someone tries to duplicate a 24-digit TRN numerical sequence in different locations with the world’s best random number generating machine (generating 250 trillion random numbers per second)^28^ but without data transfer, it would take 3000 years to accidently generate the same combination of the numbers. On the other hand, since the cosmic ray muon's travelling speed (~ *c*) and the traveling path (straight) are well known, COSMOCAT can generate 2 or more identical 24-digit TRNs in different locations within one second without physically transferring the TRNs. It is known that cosmic ray muons have a random arrival time distribution: one event occurs completely independently from the occurrence of another event. Ahlen et al.^29^ evaluated 407,420 high-energy muon arrival times, and found that there were no indications of deviations or time anisotropies in nanosecond to second time scales. (2) COSMOCAT can deliver the same numerical sequences of TRNs between the aboveground facility and a concealed underground vault. Cosmic-ray muons are penetrative, and they have been applied to imaging gigantic objects such as volcanoes^30^, ocean^31^, cyclones^32^, tectonics^33^, earthquake retrodiction^34^, Egyptian pyramids^35^, the Great Wall of China^36^ as well as underground/underwater positioning^37^ and navigation^38^, and precise time synchronization^39^.

### Principle of COSMOCATS

The COSMOCATS system consists of the sender's COSMOCAT sensor, the receiver's COSMOCAT sensor, and the receiver's storage. In the COSMOCATS scheme, (1) if the sender knows the receiver at the storage site will detect the same muons, (2) if the sender knows the distance between the sender and the receiver, and (3) if the time is accurately synchronized between the sender and the receiver, then the sender can predict the muon's arrival time at the receiver's detector. Cosmokeys are defined as timestamps generated by the muons passing through both the sender's sensor and the receiver's sensor. In COSMOCATS, these timestamps (in units of ps) are used as cosmokeys. The muon's arrival timestamp (*t*) can be described by using a numerical sequence (*N*_*i*_) as follows:

1 $$t=\sum_{i}{N}_{i}\times {10}^{-i}[\mathrm{s}],$$

where *N* can have a value from 0 to 9. The numerical sequence *N*_*i*_ is used as a cosmokey. When a muon event is observed, (at either the sender's COSMOCAT sensor or the receiver's COSMOCAT sensor) timestamps are automatically issued respectively at the sender and the receiver locations. However, in general, many of these timestamps do not match between the sender and the receiver since many of the muons do not pass though both of the sensors. The cosmokey generation rate can be calculated from the frequency of the muons which pass through both the sender's sensor and the receiver's sensor (*f*_μ_):

2 $$R_{{{\text{CK}}}} = f_{\upmu } = \Phi S,$$

where *S* is the effective detection areas of COSMOCAT sensor and *Φ* is the flux of the muons detected both by the sender's COSMOCAT sensor and the receiver's COSMOCAT sensor. The cosmokey generation rate is:

3 $$R_{{{\text{CK}}}} \sim IS^{{2}} D^{{ - {2}}} ,$$

where *I* is the intensity of the muons arriving from the zenith angle *θ*_0_, and *D* is the distance between the sender's sensor and the receiver's sensor. In the prior work, single cosmokeys were used as encryption keys. However, in order to attain sufficiently strong keys, it would be better to combine several cosmokeys to generate longer encryption keys. In the following part of this section, the methodology to connect cosmokeys is discussed. The timestamps are recorded in the receiver's sensor at the detector's single counting rate (*f*_0_) and are eventually transferred and stored in storage also at this rate. Then, a square of the ratio:

4 $$G_{{1}} = R_{{{\text{CK}}}} f_{0}{^{{ - {1}}}} ,$$

can be defined as the cosmokey matching rate between the sender and the receiver. Due to temporal jitters of the signals outputted from comparators and frequency fluctuations in clocks used in COSMOCAT, generated cosmokeys do not always match between the sender and the receiver. Therefore, the factor (*r*_CK_) coming from this effect needs to be considered, also. Consequently, the actual cosmokey matching rate is:

5 $$G_{{2}} = r_{{{\text{CK}}}} G_{{1}} = r_{{{\text{CK}}}} R_{{{\text{CK}}}} f_{0}{^{{ - {1}}}} ,$$

Due to fluctuations in time measurements, the length of each cosmokey ranges from four to six digits. TRN sequences with 15 digits to 40 digits are required in order to generate sufficiently strong encryption keys (48 bit to 128 bit). For this purpose, several cosmokeys should be combined. Therefore, the actual keys used for encrypting data would be a numerical sequence of cosmokeys: {*t*_1_, *t*_2_, …*t*_*n*_}. However, the keys combined at the sender's detector and the keys combined at the storage detector generally don’t match since *R*_CK_ < *f*_0_. Therefore, the storage user (sender) needs to encode the data for *N*_TRIAL_ times, where *N*_TRIAL_ is the number of trials required to independently generate the same encryption key between the sender and the receiver, and given by *N*_SENDER_ x* N*_RECEIVER_, where:

6 $$N_{{{\text{SENDER}}}} = N_{{{\text{RECEIVER}}}} = nG_{{2}}{^{{ - {1}}}} !/n!\left( {nG_{{2}}{^{{ - {1}}}} - n} \right)!,$$

and where *n* is the number of combinations of the generated cosmokeys required to generate encryption keys. Here it was assumed that *S* is the same for the sender and the receiver. In other words, one key out of *N*_TRIAL_ keys can be used as an encryption key. For example, if *G*_2_ is 0.2, and *n* = 3, then the sender and the receiver would respectively need to encode the data around 5 × 10^2^ times so that one out of ~ 2 × 10^5^ trials would match the key generated in the storage.

A more detailed procedure will be described in the following section by introducing an example case, but the basic concept of encoding, key storing, and authentication will be outlined here for the purpose of explaining the experimental results. The procedures the storage user needs to follow are:

A. Encode the data for *N*_trial_ times with the timestamps (*N*_*i*_ (*t*_0_)) generated at *t* = *t*_0_. The encoding rate is *f*_0_^−1^.
B. Every time the key is used for encoding, this key is erased.
  Meanwhile, in the storage facility,
C. Other timestamps are generated at a rate of *f*_0_^−1^.
  The authentication procedure for the storage user is as follows:
D. Send a set of *N*_trial_ encoded data to the storage facility.

As was described, if we can improve the timing accuracy, we can reduce *n*; hence we can drastically reduce *N*_trial_. Consequently, the key generation rate in storage could be drastically upgraded. Since the comic muon flux cannot be changed, *G*_2_ can be increased by improving a geometrical configuration and detection efficiency of the COSMOCAT system.

### Hardware and software components and the testing environment

During the current timing measurements, naturally occurring cosmic-ray muon events are detected. In order to confirm the performance of the key generation with CFD, the muon's time of flight (TOF) was measured for different distances. The current experimental setup consists of three plastic scintillators (ELJEN 200), three photomultiplier tubes (PMTs: HAMAMATSU R7724), three high voltage power supplies (0–2000 V), three CFDs (KAIZU KN381), time to digital converters (TDC: ScioSence TDC-GPX), and a field programmable gate array (FPGA: Intel MAX 10). Scintillation photons are generated in the plastic scintillator and then travel through an acrylic light guide to be processed by the photomultiplier tube. The PMT signals are transferred to CFDs via RG 50 co-axial cables. These PMT signal pulses were discriminated by CFDs in order to reduce the temporal jitter with this process, and were converted to NIM level pulses. These NIM level signals were transferred to TDCs. All of the electronics were powered by 100-Vac commercial electricity.

Three scintillation detectors (Detector A, Detector B and Detector C) were placed vertically to track vertical muons. Since muons arrive only from the upper hemisphere, with this detector configuration, the muons always pass through Detector A first, Detector B second, and Detector C third. The discriminated signals from Detector A were fed to the TDC as the start signal, and coincidence signals of those from Detector B and Detector C were fed to the TDC as the stop signal via RG-50 co-axial cables to avoid any problems associated with the frequency fluctuations of the local clock. A firmware written in FPGA processes the TDC data and outputs the hexadecimal timing data. Since the time resolution of this TDC is 27.4348 ps, the backend software convert these data to decimal data and multiply 27.4348 ps to derive the TDC time spectrum.

### Parameters used for evaluation

The value of high voltage applied to these PMTs was 1500 V. Two different spatial intervals (*D* = 120 cm and *D* = 240 cm) between Detector A and Detector C were tested, and in this interval, a lead block with a thickness of 18 cm (equivalent to a concrete slab with a thickness of 1 m) was inserted for demonstration. The spatial interval between Detector B and Detector C was fixed to be 3 cm. In order to reduce the accidental coincidence rate, only triple coincidence events between Detectors A, B, and C were considered in this work. The coincidence time window was set to be 100 ns. Considering the current detector's single count rate (~ 4 Hz), the accidental rate will be reduced to 10^–12^ Hz which is negligible for the current purpose.

**Figure 1:**
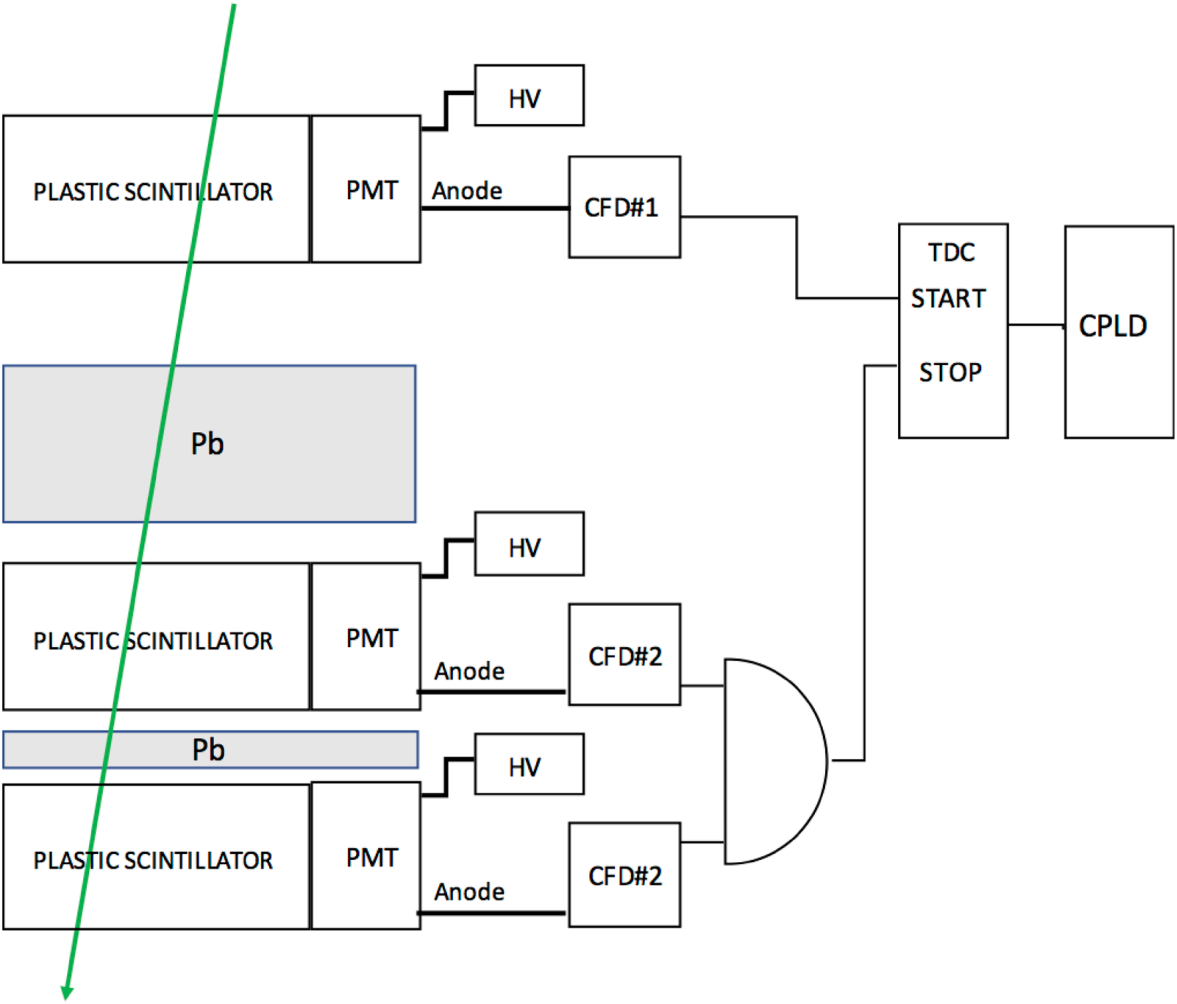
Block diagram of the experimental setup for the COSMOCATS timing evaluation. PMT, HV, CFD, TDC, CPLD and Pb respectively stands for a photomultiplier tube, a high voltage supply, a constant fraction discriminator, a time to digital converter, a complex programmable logic device, and a lead plate.

The size of the scintillators used in the current work was 20 × 20 cm^2^ with a thickness of 2 cm. The solid angles formed by Detector A and Detector C were respectively 28 msr and 7 msr for *D* = 120 cm and *D* = 240 cm. Travel distances of the photons in the scintillator (*λ*_*i*_) tend to vary as a function of time since the distance between the PMT and the muon's hitting point within the scintillator varies for each event. Therefore, the maximum time and the minimum time between the moment when the scintillation photons arrive at the sender's photocathode and the moment when the scintillation photons arrive at the receiver's photocathode are respectively:

7 $$[Dc^{{ - {1}}} + \lambda c_{\nu}^{{ - {1}}} ]_{{{\text{MAX}}}} = \left( {D^{{2}} + {2} \times W^{{2}} } \right)^{{{1}/{2}}} c^{{ - {1}}} + {1}.{4}Wc_{n}^{{ - {1}}}$$

8 $$[Dc^{{ - {1}}} + \lambda c_{\nu}^{{ - {1}}} ]_{{{\text{MIN}}}} = Dc^{{ - {1}}}$$

where *c*_*ν*_ is the speed of light in material with a refractive index of *ν* (*c*_*ν*_ = *c*/1.49 for a plastic scintillator). If we employ the parameters used for the prior work (*W* = 1 m and *D* = 70 cm), the values for [*D*(*τ*)*c*^−1^ +  *λ*(*τ*)*c*_*ν*_^−1^]_MAX_ and [*D*(*τ*)*c*^−1^ + * λ*(*τ*)*c*_*ν*_^−1^]_MIN_ are respectively ~ 12.2 ns and ~ 2.3 ns; hence, in the previous geometrical configuration, there was an uncertainty of timing of ~ 10 ns at the most. On the contrary, the setup employed in the current work (*W* = 20 cm and *D* = 120 cm and 240 cm) reduces this uncertainty to, at most, a value of ~ 1 ns. The block diagram designed for the current experiment is shown in Fig. 1.

### Results and the significance of the improvements observed

As was described in the "Principle of COSMOCATS" subsection, the accuracy of the time recorded by the detector determines the number of timestamps we need to use to generate encryption keys. In this subsection, a detailed analysis results and how the proposed algorithm works are described in a step by step manner, and the significance of the improvements is shown.

**Figure 2:**
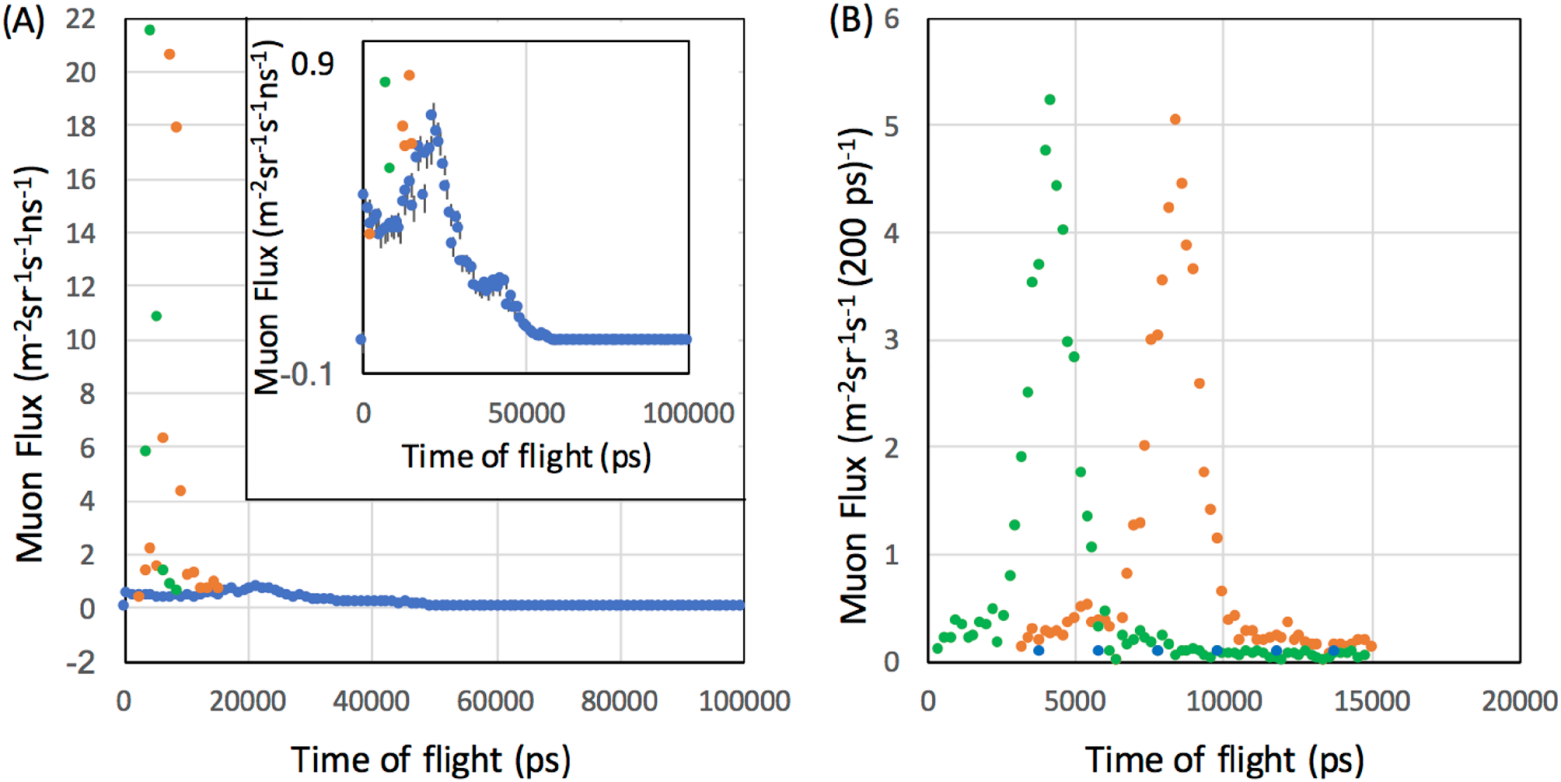
Comparison of the time of flight distribution between the prior work and the current work. The time spectrum as obtained in the prior work is indicated with blue filled circles. The time spectra as obtained in the current work for a distance of 120 cm (green circle) and 240 cm (orange circle) are overlaid (**A**). The magnified view in the temporal region between 0 and 20 ns is shown (**B**). The error bars are within the circles. The data of the prior work were taken from Tanaka^26^.

A. First, the TDC time spectra were generated to compare the muon's TOF for different spatial intervals between Detector A and Detector C (*D* = 120 cm and *D* = 240 cm). The distribution of the time displacements (*t*_RECEIVER_ − *t*_SENDER_) observed Detector A and Detector C are shown in these TDC spectra. The time required for muons to travel 120 cm and 240 cm are respectively 4 ns and 8 ns.
B. Next, the shapes of these 2 TDC time spectra were examined. As a result, the shapes of both of these TDC spectra showed almost identical unimodal Gaussian-like shapes (Fig. 2). The standard deviation of these spectra was calculated to derive the timing accuracy. The values were 4.2 ± 0.9 ns and 8.4 ± 0.9 ns for *D* = 120 cm and *D* = 240 cm, respectively.
C. The currently obtained TDC spectra were compared with those measured in the prior work. There, the spatial interval between Detector A and Detector B was 70 cm. There are two major differences between the current work and the prior work. (1) The peaks measured in the prior work are much broader than the peaks we measured in the current work. This broadness comes from the jitter of the comparators used in the prior work. (2) While the time required for muons to travel 70 cm was 2.3 ns, there were three peaks at ~ 2 ns, ~ 20 ns, and ~ 45 ns where the 20-ns peak was largest, and the other two peaks were much smaller than this peak (Fig. 2A inset). This large offset was due to the short-time scale drift effect of the GPS-DO.

As a consequence, the accuracy of timing muon arrivals was improved by two orders of magnitude (from ~ 100 to ~ 1 ns). In conclusion, it was confirmed that there were significant improvements in timing in terms of offset and precision of timing muon arrivals. Therefore, the algorithm proposed in the "Principle of COSMOCATS" subsection works more efficiently for the following reasons.

A. In the prior work, as a consequence of discrepancies in TOF, the time series of *N*_3≤*i*≤6_ (4 digits) were used as cosmokeys. In the current work, the time series of *N*_3≤*i*≤8_ (6 digits) can be used as cosmic keys.
B. In order to generate encryption keys with 24 digits (128 bit), with 4-digit cosmokeys and 6-digit cosmokeys, *N*_trial_ would be respectively 593,775 and 4845 for a given *G*_2_ = 0.2 with the prior and current COSMOCAT system. Therefore, encryption key generation rate is increased by more than 2 orders of magnitude.
C. As shown in Eq. (6), the encryption key strength and the encryption key generation rate are in the tradeoff relationship. For example, 24 decimal digit keys (*n* = 4 for 6-digit cosmokeys) are 10^4^ times stronger than 20 decimal digit keys (*n* = 5 for 4-digit cosmokeys). (B) could be rephrased that the current setup can generate stronger keys than the prior setup in a unit time.

*f*_μ_ values observed for these distances (120 cm, and 240 cm) were respectively ~ 0.1 Hz and ~ 0.02 Hz. Since the value of *G*_2_ depends on the broadness of the TOF spectrum, *N*_trial_ can be further improved and will be discussed in the following paragraph.

**Figure 3:**
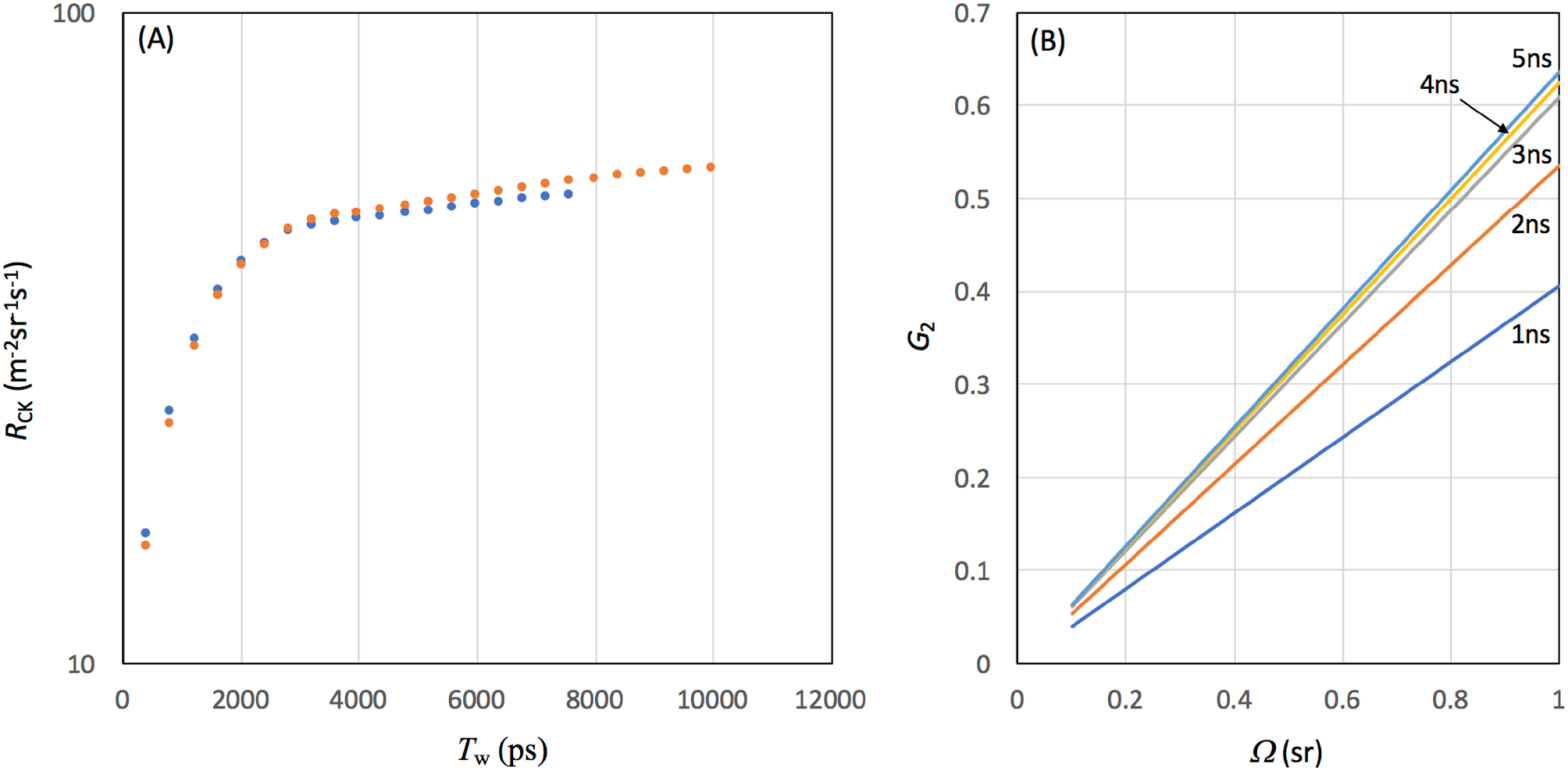
Cosmokey generation rate as a function of the time window. The data points are shown with blue (*D* = 120 cm) and orange (*D* = 240 cm) filled circles (**A**). The actual cosmokey matching rate is also shown as a function of the solid angle formed by the sender's sensor and the receiver's sensor for different time windows (**B**).

*R*_CK_ can be derived by integrating the time spectrum (blue filled circles) shown in Fig. 2 over the time range between 0 and the given time window (*T*_W_) such that:

9 $${R}_{\mathrm{CK}}={\int }_{0}^{{T}_{\mathrm{W}} \mathrm{ns}}f(t^{\prime})dt^{\prime}$$

where *f*(*t*′) is the event frequency at *t*′. The value of Eq. (9) for *T*_W_ = 100 ns was 20 m^−2^ sr^−1^ s^−1^ in the prior work. Figure 3A shows *r*_CK_*R*_CK_ as a function of *T*_W_ (120 cm and 180 cm) measured in the current work. There was no large distant-dependent difference in *r*_CK_*R*_CK_. For* T*_W_ = 1 ns, *r*_CK_ = 0.58 for 120 cm and *r*_CK_ = 0.49 for 240 cm; for *T*_W_ = 3 ns, *r*_CK_ = 0.87 for 120 cm and *r*_CK_ = 0.77 for 240 cm. Figure 3B shows *G*_2_ as a function of *Ω* for different *T*_W_. Consequently, *N*_trial_ could be drastically reduced. For example, if we compare *N*_trial_ for the same geometrical configuration with the prior work (*Ω* ~ 1sr), the *N*_trial_ values required for the sender to share encryption keys in the storage with digits of 20 digits in the prior work and 24 digits in the current work are respectively 53,130 and 70.

## Discussion

### Potential limitations-scalability

The key generation rate increases as a function of the detector size. However, as shown in Eqs. (7) and (8), the key strength is reduced due to uncertainties coming from the detector size. In order to solve this problem, it is necessary to modularize the detector within the unit size that mustn't exceed the given allowance of timing uncertainty, and to increase the number of modules. Since the tracking rate is reduced as a function of the distance as shown in Eq. (3), the key generation rate is limited not only by the size of the detectors but also by the distance between detectors.

### Potential limitations-hardware requirements

COSMOCAT doesn't include any critical device for safety requirements. Plastic scintillators are non-inflammable. The electric current generated by the high voltage power supply is measured in the sub-milliamps scale. While in the current experimental setup, all of the detectors and electronics are wired. This limits potential availability of COSMOCATS since for example, the underground safe or submarine and the aboveground device must be wired. However, a non-wired alternative solution using cosmic time calibrator (CTC) will be discussed later in this section.

### Potential limitations-environmental factors affecting muon detection

As was mentioned in the previous section, since cosmic-ray muons are highly energetic, the key generation rate is not strongly affected by variations in ambient temperature and electromagnetic field. However, the muon rate is reduced in the underground/underwater environments. For example, it is reduced by 99% at 100 m and by 99.999% at 1000 m^40^. Therefore, the key generation rate is significantly reduced in deep underground/underwater environments.

### Potential vulnerabilities that may still be present

If eavesdroppers were to setup an additional detector above or underneath COSMOCATS, and if they know the time-zero moment defined by COSMOCATS, they will be able to steal keys. However, this risk can be mitigated by frequently changing the time-zero values. Since these values are used only for time synchronization, these values don't have to be known by users and thus, security of these values is somewhat guaranteed.

### Authenticating rate

As was described in the previous section, in order to authenticate the sender's data with the key stored in storage, the storage user needs to send a set of *N*_trial_ encoded data to the key storage facility. In the key storage facility, the *N*_trial_ encoded data will be authenticated by verifying the *N*_storage_ timestamps. If the detector size of the sender's COSMOCAT sensor and that of the receiver's COSMOCAT sensor are the same, and if the data encryption at the sender and the key generation at storage are conducted in the same time period, *N*_storage_ = *N*_trial_. In the prior work, 53,130 × 20 digits = 1,062,600 patterns in the encoded data need to be verified (to find a match) with the 1,062,600 patterns of the keys stored in storage. The time required to verify 10^12^ patterns is 25 s, and thus the authentication rate limit would be 0.04 per second with a currently commercially available graphic card: Gigabyte GeForce RTX 2080 Ti Turbo 11 GB Graphics Card^41^. However, if we use *N*_trial_, a value achieved by the current work, the number of patterns we would need to verify would only be 3 × 10^6^ patterns for 24 digit keys; hence the authentication rate limit would reach a rate of 12,000 per second. This is an improvement by more than 5 orders of magnitude.

### Time synchronization scheme

Degradation of the cosmokey length due to temporal jitters of the signals outputted from comparators was solved. In order to replace GPS with another device, we need an alternative wireless ns-level time synchronization scheme. However, the currently available wireless time synchronization technique is RF-based and offers only a microsecond-order time synchronization precision^42,43^. Not only does RF fall short of the precision requirements of COSMOCAT, but also RF cannot penetrate matter sufficiently to reach the underground safe location.

Recently, the cosmic time synchronizer (CTS) was developed^39^ as a wireless underground/underwater time synchronization technique, but the time synchronization precision was limited to ~ 100 ns. The easiest way to synchronize the sender's clock and the receiver's clock at the storage site is by wiring these clocks. However, if this strategy was used for COSMOCATS, the remaining physical traffic between the sender and the storage may reduce the security level of the system. Therefore, it is better to keep the storage physically separated at a distance at least while encoding files on the server and generating encryption keys in the storage. Therefore, the cosmic time calibrator (CTC) scheme is employed in this work.

**Figure 4:**
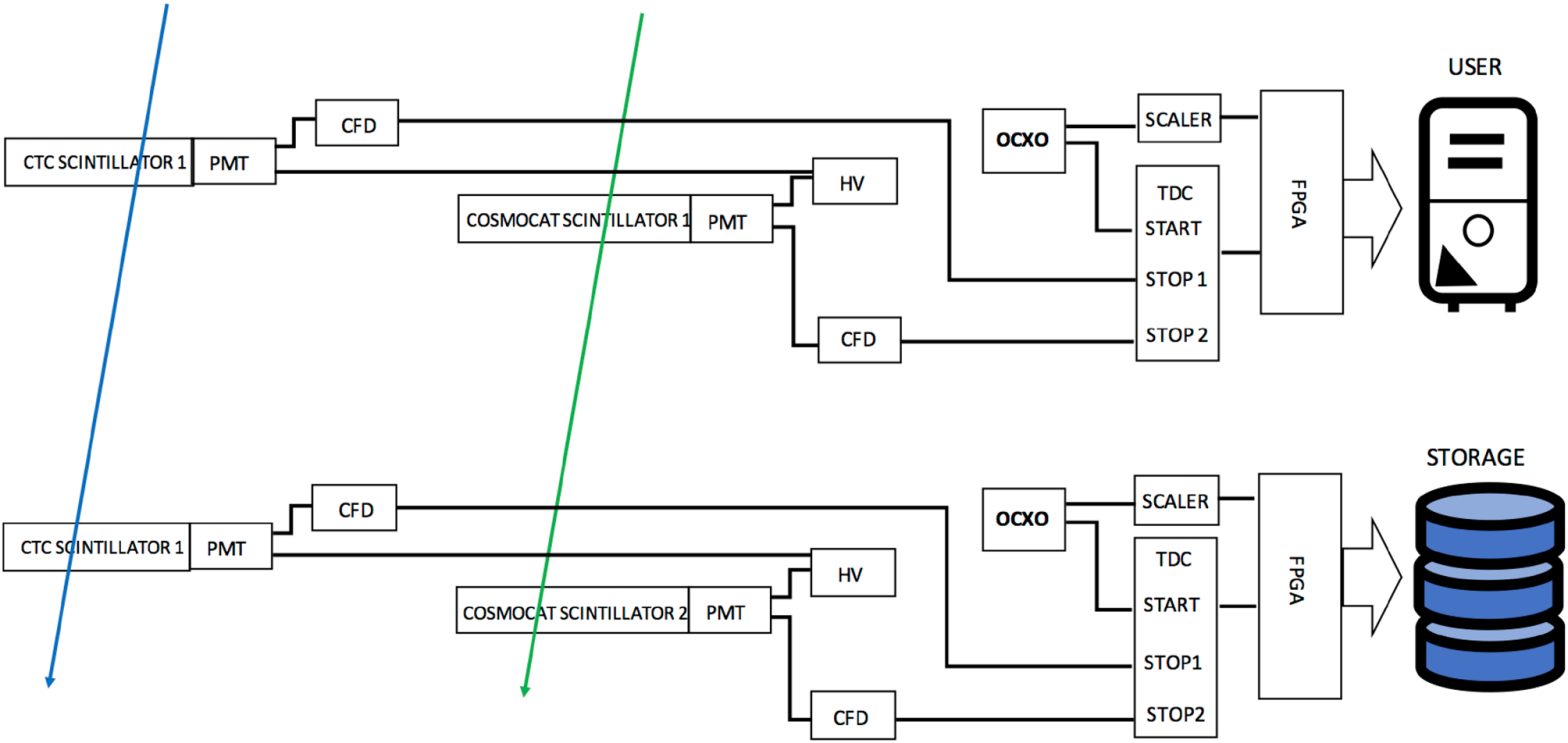
Block diagram of the CTC-based COSMOCATS system. The notation in this diagram is the same as the one used in Fig. 1. The blue and green arrows indicate the different muons.

Figure 4 shows a diagram of the setup for the CTC-based COSMOCATS system. In this system, COSMOCATS timestamps are issued by local oscillators called OCXOs (oven controlled crystal oscillators). Since the frequency of OCXO outputs drifts in a different way between the sender's OCXO and the receiver's OCXO, the timestamps issued by the same muons could be recorded as different timestamps between the sender's OCXO and the receiver's OCXO. However, in this system, this difference is retrospectively corrected with another pair of muon sensors that are prepared in the vicinity of the COSMOCATS system.

CTC uses cosmic ray muons as calibration signals to correct local clocks associated with COSMOCAT sensors. If the sender's local clock is defined as the standard clock (labeled as Clock 0) then its successive clock is named here as Clock 1. The times measured by Clock 0 and Clock 1 are respectively labeled with *τ* and *t*. Since most of a cosmic-ray muon's Lorentz factor is much larger than 1, it is reasonable for us to approximate the time (*T*) required for cosmic-ray muons to travel a distance between Detector 0 and Detector 1 to be *Dc*^−1^. Consequently, if a cosmic-ray muon passes through Detector 0 and Detector 1, then the moment when the muon has passed through Detector 1 is measured by Clock 1 (*t*) as:

10 $$t = \tau + Dc^{{ - {1}}} + \delta \tau$$

**Figure 5:**
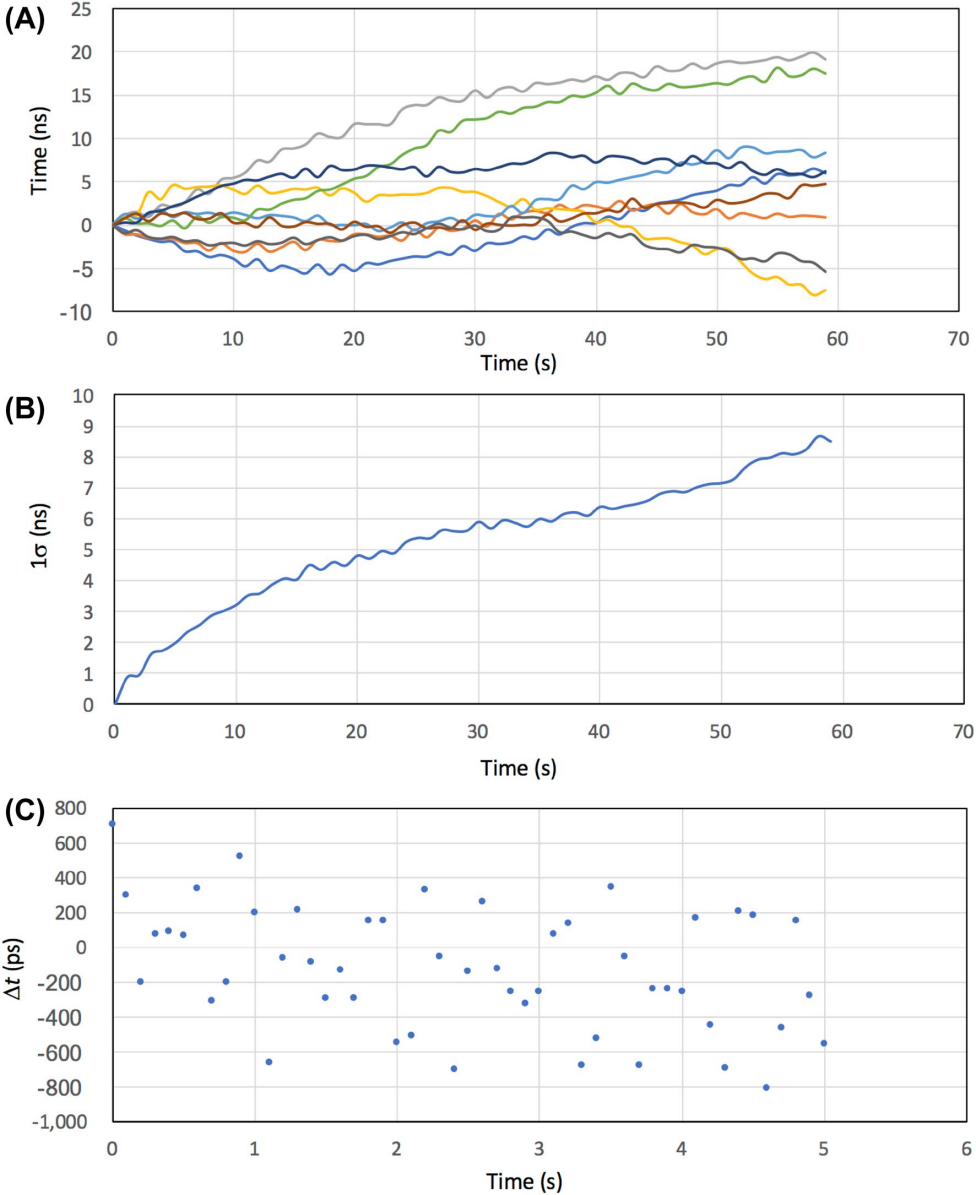
Temporal fluctuations in CTC with OCXO. Fluctuations for 9 independent runs are shown as a function of time (**A**). A standard deviation calculated from 9 independent runs is shown as a function of time (**B**). A magnified view of 10th run within the time range between 0 and 5 s is also shown (**C**).

*δτ* comes from the relative drift of Clock 1 measured with Clock 0. Since *D* is known, *δτ* can be derived from *t* and *τ*. The information of *τ* can be associated with the data when the sender encodes the data with the COSMOCAT system, and can be sent to the storage receiver when the senders authenticate their encrypted data. The muon rate is limited, and the OCXO doesn't drift so far within a short period of time (Fig. 5A). In order to remove effects from the comparators/geometrical configuration of the setup, signals from a clock generator (Technoland N-TM 715) split with a fan-out circuit (Technoland N-TM 605) instead of signals from the scintillation detectors were fed into TDC shown in Fig. 4. In Fig. 5B, a standard deviation calculated from 9 independent runs of OCXO is shown as a function of time. If the CTC steering frequency is < 0.1 Hz, > 99.7% of the time stamps can be corrected by finding coincident events within a time window of *t*_W_ = 10 ns for the muon that passed through Detector 0 and Detector 1. Figure 5C shows a magnified view of temporal fluctuations acquired in another OCXO run within the time rage between 0 and 5 s with a sampling rate of 10 Hz (the data shown in Fig. 5A,B are sampled at 1 Hz). In this specific case, a standard deviation within this time range was 349 ps, indicating that CTC would work satisfactory for the COSMOCATS system.

The clock correction errors due to the accidental coincidence can be neglected. As can be seen in Eq. (11), the time window set for finding coincidence events is generally much shorter than the open-sky muon's arrival rate (*f*_0_^−1^):

11 $$t_{{\text{W}}} < < f_{0}{^{{ - {1}}}} .$$

Therefore, the accidental coincidence is negligible if we use four CTC sensors, and if we look for fourfold coincidence events such that:

12 $$T = 0.{25}t_{{\text{W}}}{^{{3}}} f_{0}{^{{ - {4}}}} ,$$

where *T* is the accidental coincidence interval. For example, if *t*_W_ = 10^–8^ s, and *f*_0_ = 10^2^ Hz, the accidental coincidence would occur every 2.5 × 10^15^ s. The following procedure describes the CTC process: (A) TTL pulses outputted from OCXO are continuously counted by the scaler respectively at Detector 0 and Detector 1. These count numbers are respectively defined as *N*_0_ and *N*_1_. (B) Once *τ* information (*N*_0_) is sent to Clock 1, *Dc*^−1^+* δτ* is calculated from Eq. (11), and it is then subtracted from *t* (*N*_1_)*.* By repeating this process, Clock 0 and Clock 1 can be resynchronized in a retrospective way with a precision of <  < 10 ns as is indicated in Fig. 5B,C. The correction time intervals are equivalent to the key generation time intervals and the authenticating time intervals that are generally much shorter than 5 s.

### Secure electronic digital signing

The caveat of the COSMOCATS system is that the storage is invincible only when files are being encoded on the user's server and encryption keys are being generated in storage. However, there are several useful applications of this storage system which can be used to generate an electronic digital signature. The electronic digital signature system is an essential concept in modern society: integral to cryptocurrency, e-commerce websites, social media, banking apps and any other exchange of sensitive data. Here we consider a model of cryptocurrency using COSMOCAT and issued by a fictitious organization called COSMOBANK as an example. In this scenario, storage is located inside the underground safe of this bank. This underground safe is built in the COSMOBANK building and there is no entrance; hence no human can access the underground safe. Therefore, this safe is physically protected. The cryptocurrency called cosmocurrency is issued by COSMOBANK. Cosmocurrency functions like cash and encrypts the data showing its value (10 USD, 100 USD, etc.) with the COSMOCATS system equipped for COSMOBANK. The key used for encrypting each cosmocurrency is used as an identification number for each cosmocurrency “bill”.

### Issuing process

The procedure to issue the cosmocurrency is as follows. The aboveground COSMOCAT sensor encrypts the cosmocurrency data and the encrypted cosmocurrency is transferred to the COSMOBANK server that is connected to the internet. At the same time, the underground COSMOCAT sensor located inside the safe records the timestamps and transfers them to storage. CTC is used for recording the time calibration information which is later used for the authentication process. Neither physical nor cyber connections exist between the aboveground server sensor to storage. Therefore, at this stage, the identification numbers (24-digit encryption keys) of the cosmocurrency cannot be leaked. Also, since cosmocurrency is strongly encrypted in this process, any spying third party that managed to access the cosmocurrency data (protected by 24-digit keys) doesn't have a possibility of tampering with the cosmocurrency. (It would take approximately one million years to crack with a computer using a currently commercially available graphic card: Gigabyte GeForce RTX 2080 Ti Turbo 11 GB Graphics Card^41^).

### Authenticating process

The authenticating process would be required when a transaction is performed with cosmocurrency. At this stage, there are physical connections between the COSMOBANK server and the underground storage. The physical connection for uploading from the COSMOBANK server to the underground storage is one way. The device that can be used for this purpose is e.g., a USB-based DataBridge^44^. A DataBridge is a security device that connects terminals with a USB cable and allows one-way data transfer only while the terminals are connected. Therefore, it is impossible to download the keys from the storage on the server's side. Even if the data bridge itself is cracked, the data on this traffic is already highly encrypted. The physical connection for downloading data from this storage is an RG50 coaxial cable. If the data are successfully authenticated by the key in the storage, (A) the key in this storage is erased, and (B) a TTL pulse is outputted to the COSMOBANK server. After this authentication process, COSMOBANK will know the corresponding cosmocurrency is the authentic currency.

**Figure 6:**
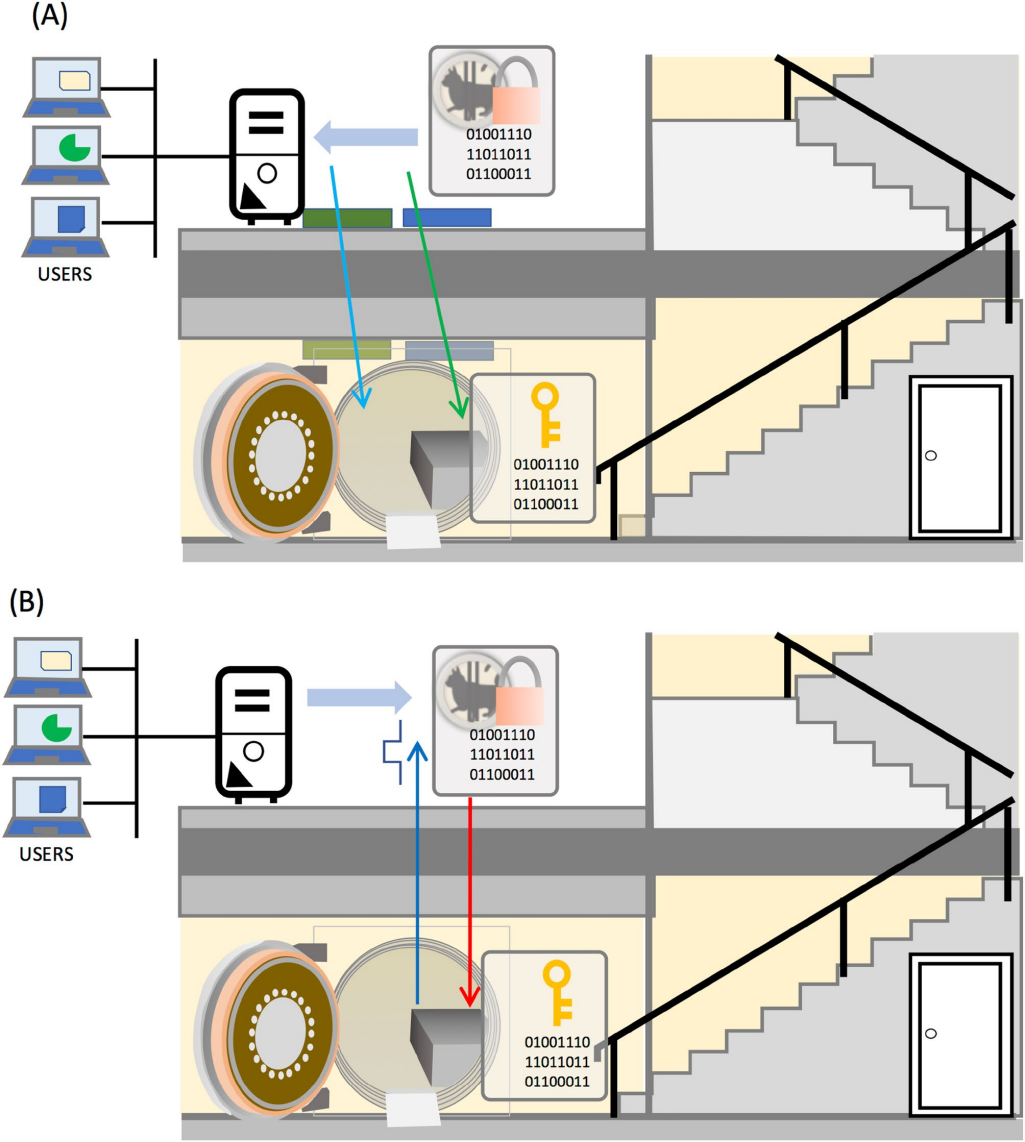
Scheme of COSMOCATS-based key generation and authentication. The cosmocurrency issuing (**A**) and authenticating (**B**) processes are shown. In this scheme, a data storage is located inside an underground safe. Blue and green boxes respectively indicate the COSMOCAT sensors and CTC detectors. Blue and green arrows indicate muons from different origin. Each cosmocurrency (box with a coin, lock and numbers) contains the information about its value to be decrypted by each key assigned to each cosmocurrency (box with a key and numbers). Red and black arrows respectively indicate the one-way traffic through a USB-based data bridge and an RG50 coaxial cable.

This authenticating process guarantees the security of cosmocurrency for the following reasons: (A) the cosmocurrency users cannot duplicate this currency since the same cosmocurrency used for the second transaction cannot be authenticated, and (B) eavesdroppers would get no useful data since information related to encryption keys is not included in this TTL pulse. The cosmocurrency issuing and authenticating processes are summarized in the diagram shown in Fig. 6. The aforementioned protocol indicates that once the encrypted data like cosmocurrency are generated, these can be used not only for virtual currency but also for other electronic digital signing processes on other occasions for other purposes.

The majority of other cryptocurrency uses digital signatures and cryptocurrency mining to store data on a public ledger that cannot be altered retroactively without altering all subsequent blocks: blockchains that use the hashing algorithm. As security and privacy of a blockchain solely depends on the hashing and digital signatures, a number of researchers^45–51^ have discussed about how the fast progress of quantum computing has opened up the possibilities of attacks on blockchains through Grover’s and Shor’s algorithms. ECDSA is mainly used as a way to sign digitally. It uses the logarithmic problem for security which cannot be solved by classical computers, making it secure for now, but this security will be compromised with the advancement of quantum computing. Moreover, the most criticized issue of this system is excessive energy consumption^52^. There is a report that the amount of energy consumed by bitcoin mining exceeds the total electricity consumed in dozens of countries^53^. It is clear that the mining process will have various environmental consequences due to the excessive amount of energy consumption required. By following the aforementioned COSMOCAT-based protocol, not only would energy requirements be reduced, but also the safety of issuing currency and transactions will be guaranteed and thus, centralized cash management will be possible. As a result, the standard public ledger distributed within a peer-to-peer computer network can be discarded; hence the mining process can be removed from cryptocurrency.

### Secure communication

**Figure 7:**
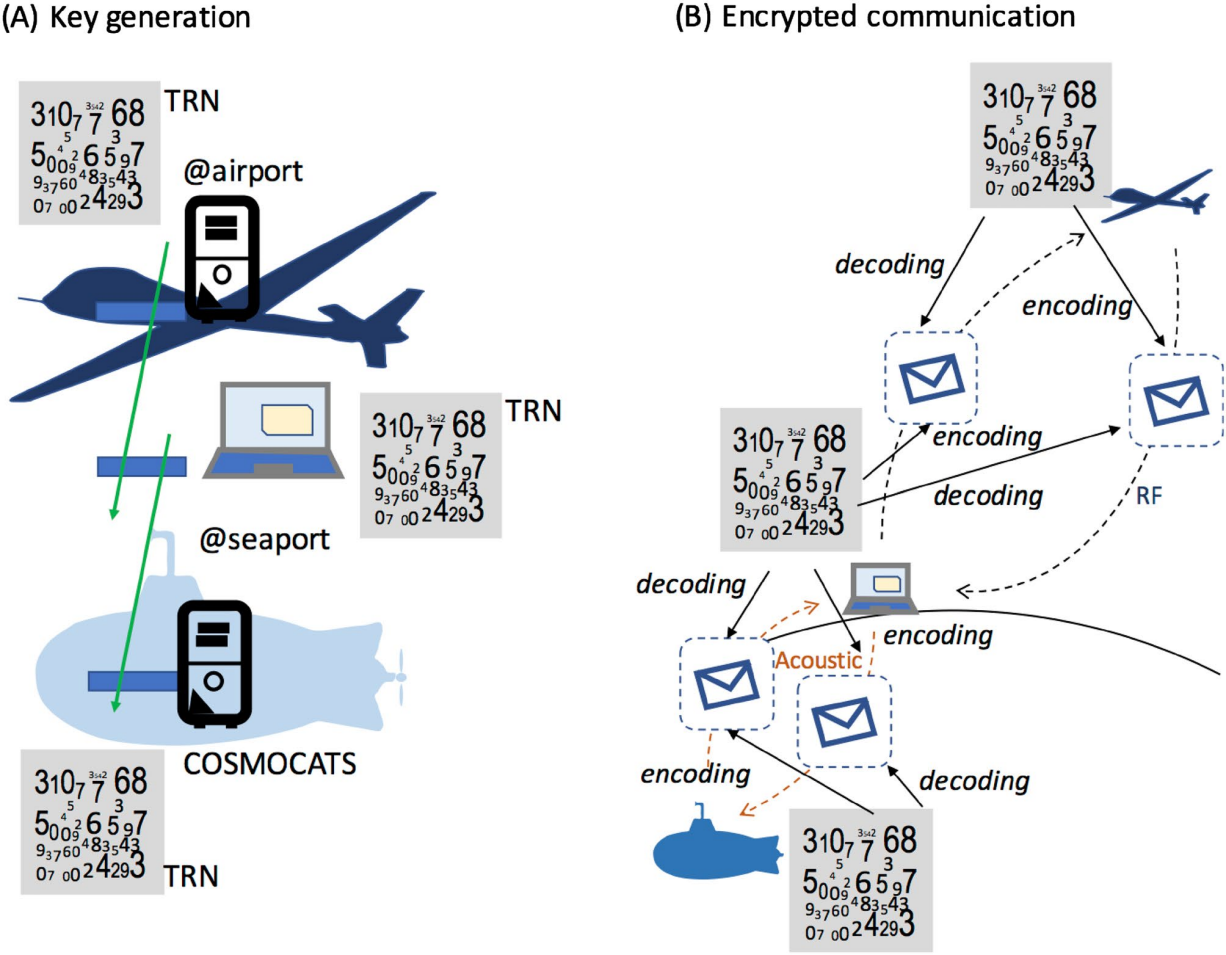
Applications of COSMOCATS to secure wireless communications to various vehicles. Encryption keys are generated in both the user's device and the vehicles by utilizing true random numbers (TRN) generated by muons (**A**). When the users send the messages to these vehicles, the users encode their data with these encryption keys to transfer their messages to the vehicles with the conventional signal transfer techniques such as RF and acoustic techniques, and these messages are decoded by utilizing the encryption keys duplicated in these vehicles (**B**). The same procedure is applied also when these vehicles send the messages to the users.

Rapid growth of future secure wireless communications to various vehicles are expected. The communication target ranges from unmanned aerial vehicles (UAVs)^54^ to autonomous underwater vehicle (AUV) and submarines^55^. The COSMOCATS system has the capability to establish highly secured wireless communication between land-based users and these vehicles. The process would be as follows: before a UAV, AUV or submarine departs from an airport or seaport, users generate keys both in the user's device and the storage in these vehicles. While keys are generated, the user's device is totally isolated from the network. Therefore, keys exist only in COSMOCATS or in the user's device. Users later could use these keys to encode their data and send them to AUVs/UAVs/submarines with a regular communication methodology (Fig. 7). When these vehicles receive the encrypted data, they are decoded by using keys stored in the storage. Since there are no key exchanges between the users and vehicles from the beginning to the end, the communication contents cannot be eavesdropped. The only caveat would be that users need to be sufficiently close to the vehicles when keys are generated.

### Secure cloud storage

Storing data remotely to the cloud in a flexible on-demand manner brings various benefits, but there is a potential risk. A cloud data storage service usually involves the cloud user, the cloud service provider (CSP) and the third-party auditor (TPA). Users rely on the CSP for cloud data storage and maintenance. However, for their own benefits the CSP might neglect to keep the prescribed protocol execution. We assume that the TPA is reliable and independent. However, the TPA could learn the outsourced data after the audit. If, alternatively, the data are all encrypted with COSMOCAT and the keys are stored in COSMOCATS which is located where the TPA cannot physically access, the TPA will not have the opportunity to eavesdrop on the user's data.

### Future research directions

The future development of more advanced timing techniques will improve efficiency of key generation and distribution that could further enhance security. Although picosecond-level timing resolution is not easy to achieve in particular with a relatively large detector, several applicable techniques have been reported using Chrenkov radiation. For example, timing resolution of 50 ps and 25 ps have been respectively attained with Belle II Time of Propagation particle identification system^56^ and Micromegas detectors^57^. The future research and development studies include investigating the possible high precision timing technologies for the requirements of a COSMOCATS upgrade.

As was discussed in Introduction section, security research (such as authentication) is not incorporated into QKD research, and QKD itself cannot be used to protect data storage. On the other hand, COSMOCAT realizes secured data storage. However, there are spatial limitations on the range of COSMOCATS (e.g., the user must have access right above or right beneath the COSMOCATS), for information transmitted on the network by the users to be totally secured. Therefore, a profitable direction for future research can also include utilizing a hybrid combination of both QKD and COSMOCATS, to make the most of each technique’s strengths and to mitigate each technique’s weaknesses, in order to upgrade the security and maintenance capabilities of network services.

In conclusion, it was shown that COSMOCAT has the potential to realize invincible key storage that enables ultra-high security electronic digital signing. While the advantage of symmetric-key cryptosystems is that they are invulnerable against quantum computers and that processing is faster than public-key cryptosystems, the disadvantage is that it requires careful handling of keys. This is because no matter how complicated the encryption is, if the key is stolen, anyone can decrypt the data. Since the encryption side and the decryption side must have the same key, the possibility of key leakage increases as the number of holders increases. The COSMOCATS system has solved these problems. The COSMOCATS system takes advantage of the two major and unique features of COSMOCAT: strong penetrability to underground locations and the capability to generate identical multiple TRN sequences at a distance without having to physically transfer these sequences. Consequently, we can construct a new symmetric-key cryptosystem between aboveground locations and concealed underground locations. It is anticipated COSMOCATS will contribute towards the establishment of a new system for postquantum electronic digital signing, including cryptocurrency, that doesn't require blockchains; hence helping ensure unprecedented levels of security for users and cutting energy consumption problems associated with other techniques.

## Data Availability

The datasets used and/or analyzed during the current study are available from the corresponding author on reasonable request.
